# Rapid Eye Movement (REM)-Predominant Central Sleep Apnea With Alpha-Delta Sleep Pattern: A Case Report

**DOI:** 10.7759/cureus.86702

**Published:** 2025-06-24

**Authors:** Rafeek Kandy, Lubna Alhourani, Mateen Uzbeck, Said Isse, Ali Wahla, Zaid Zoumot, Adam Bennett, Irfan Shafiq

**Affiliations:** 1 Respiratory and Allergy Institute, Cleveland Clinic Abu Dhabi, Abu Dhabi, ARE

**Keywords:** alpha-delta sleep, central sleep apnea, cpap therapy, opioid-induced central apnea, pregabalin, rem-predominant csa, tramadol

## Abstract

Central sleep apnea (CSA) occurring predominantly during rapid eye movement (REM) sleep represents an unusual clinical phenomenon, as CSA typically occurs during non-rapid eye movement (NREM) sleep. We present the case of a 32-year-old male with severe obstructive sleep apnea (OSA), with an apnea-hypopnea index (AHI) of 65.5/hr, who exhibited central apneas mainly during REM sleep (central apnea index: 35.9/hr in REM vs. 5.2/hr in NREM), along with concurrent alpha-delta sleep pattern during slow-wave sleep. The patient was taking tramadol 100 mg and pregabalin 300 mg daily for chronic back pain. Continuous positive airway pressure (CPAP) therapy at 6 cmH₂O effectively managed the OSA and resolved both central apneas and the alpha-delta sleep pattern despite continued medication use. The CPAP therapy reduced the total AHI from 65.5/hr to 1.3/hr, with the central apnea index decreasing from 35.9/hr to 0/hr during REM sleep. This case contributes to the limited literature on REM-predominant CSA and demonstrates the efficacy of standard CPAP therapy in this uncommon presentation, suggesting complex interactions among pharmacological agents, sleep architecture, and respiratory control mechanisms.

## Introduction

Central sleep apnea (CSA) is primarily a phenomenon of non-rapid eye movement (NREM) sleep, especially during lighter stages (N1 and N2), where ventilatory control is more chemically driven and unstable. During NREM sleep, breathing regulation transitions from behavioral/voluntary control to predominantly metabolic control, removing the wakefulness drive to breathe. This shift makes ventilatory motor output primarily dependent on chemoreceptor feedback. A critical feature of NREM sleep is the emergence of a “hypocapneic apneic threshold,” where even small decreases in PaCO_2_ can result in breathing cessation [1,2].

In contrast, respiratory control during rapid eye movement (REM) sleep relies on complex neurological processes. Compared to NREM sleep, REM sleep exhibits lower chemosensitivity to hypercapnia, increased upper airway resistance, irregular breathing patterns, and is associated with relative hypoventilation [3]. Despite these variations, the central respiratory drive maintains sufficient ventilation to prevent central apneas, despite the physiological changes and blood gas fluctuations characteristic of REM sleep. Ludwig et al. (2023) [2] demonstrated that central apneas occur less frequently during REM sleep in patients with obstructive sleep apnea (OSA), implying that the reduced chemosensitivity characteristic of REM may serve a relative protective role.

Opioids reduce respiratory regulation by activating μ-opioid receptors in important brainstem areas, including the pre-Bötzinger complex (a critical brainstem region that generates the basic rhythm of breathing), which generates respiratory rhythms [4,5]. Tramadol, a weak μ-opioid agonist with additional monoaminergic properties (affecting serotonin and norepinephrine pathways), may have a different respiratory profile than potent opioids [6].

Alpha-delta sleep occurs when alpha activity (8-13 Hz) overlays delta waves (0.5-4 Hz) during slow-wave sleep, potentially indicating a disturbance in microarchitecture. It is linked to increased pain perception and psychological distress in fibromyalgia, along with morning stiffness and fatigue in rheumatoid arthritis [7,8]. However, Mahowald and Mahowald (2000) [8] noted that this pattern is nonspecific and can occur in normal individuals and various conditions with no musculoskeletal symptoms.

This case report presents an unusual combination of central apneas predominantly during REM sleep occurring concurrently with an alpha-delta sleep pattern in a patient taking medications known to affect respiratory control. Both phenomena were effectively managed with standard CPAP therapy.

## Case presentation

We report an exceptionally rare case of REM-predominant CSA in a 32-year-old male with a seven-year history of hypertension (HTN) who presented to the emergency department (ED) with worsening episodes of orthopnea, specifically occurring during sleep or in the supine position. He reported nocturnal choking episodes, prompting multiple ED visits due to a sensation of breathlessness. Despite an unremarkable cardiopulmonary workup, his persistent symptoms raised suspicion of sleep-disordered breathing. The ED physician referred him to the sleep medicine clinic for further evaluation and a diagnostic sleep study.

Upon presentation, vital signs were notable for hypertension (148/95 mmHg) and tachycardia (101 bpm). Physical examination was largely unremarkable, with no signs of acute respiratory distress, congestive heart failure, or other systemic abnormalities. Oxygen saturation was 100% on room air.

The patient was evaluated at the sleep clinic four days later, where he reported recurrent nocturnal episodes suggestive of obstructive sleep disturbance, including a sensation of choking, which had previously resulted in ED visits. The patient reported that his symptoms had begun more than a year ago. The examination highlighted a body mass index (BMI) of 28.4 kg/m^2^ and a crowded upper airway with a Mallampati score of 3. His Epworth Sleepiness Scale (ESS) was 12, indicating increased daytime somnolence, and the STOP-BANG score was 5. He had a past medical history of HTN for the last seven years and was on amlodipine. The patient was married and denied the use of alcohol, tobacco, or illicit substances but reported daily vape use. There was no relevant family history contributing to the present condition. Given the clinical presentation, a diagnostic polysomnogram was ordered to clarify the nature and severity of the suspected sleep apnea.

The patient underwent a type 1 sleep study, which showed severe OSA with a total AHI of 65.5/hr, composed of a central apnea index of 12.3/hr, an obstructive apnea index of 7.0/hr, a mixed apnea index 1.6/hr, and a hypopnea index of 44.6/hr. Significant oxygen desaturations were noted, with the lowest saturation of 55%. Sleep architecture showed a total sleep time of 306 minutes, sleep efficiency of 88.7%, N1 of 10.9%, N2 of 44.1%, N3 of 21%, and REM of 24%. The arousal index was 40.2/hr, the periodic limb movement (PLMS) index was 2.9/hr, and loud snoring was noted throughout the night.

Central apneas were predominantly observed during REM sleep, while hypopneas and obstructive apneas primarily occurred during NREM sleep. The central apnea index was 35.9/hr in REM vs. 5.2/hr in NREM, confirming the unusual REM-predominant distribution of central events. Notably, the number of central apneas increased progressively across successive REM periods, with the highest number observed during the final REM period. The patient slept in the supine position throughout the study, eliminating posture as a contributing factor. The sleep study also revealed an alpha-delta pattern, characterized by the superimposition of alpha frequency activity onto delta waves during slow-wave sleep. This pattern was observed throughout the study.

**Figure 1:**
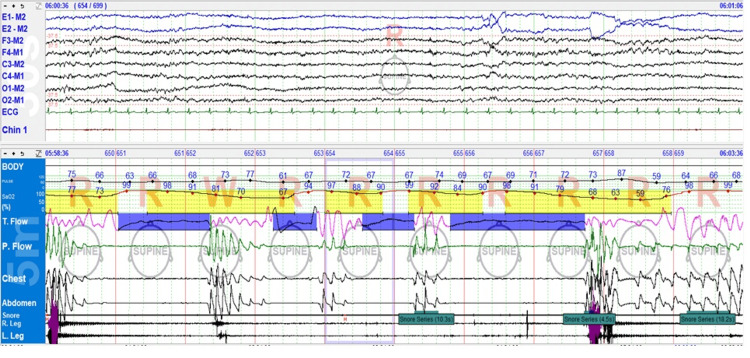
Central apnea during REM sleep during the diagnostic study. Top sleep pane shows a 30-second epoch, and the bottom respiratory pane shows a five-minute epoch. Longer central apnea events resulted in more severe oxygen desaturations, as visible in the recording. REM: Rapid eye movement.

The unusual presence of central apneas during REM sleep (Figure 1) prompted consideration of medication effects or underlying neurological factors affecting respiratory control.

**Figure 2:**
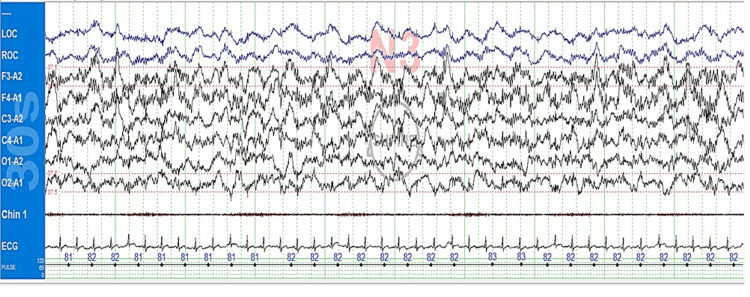
Alpha-delta sleep during the diagnostic study, 30-second epoch

The concurrent presence of alpha-delta sleep (Figure 2), often associated with chronic pain disorders, prompted inquiry about pain-relief medications. Upon checking Malaffi (United Arab Emirates (UAE's) health information exchange platform across healthcare providers), we discovered that the patient was prescribed and taking tramadol 100 mg and Lyrica (pregabalin) 300 mg daily for chronic back pain for almost two years from an outside facility. Notably, the patient had not previously disclosed this medication use during clinical evaluation in the sleep clinic.

**Figure 3:**
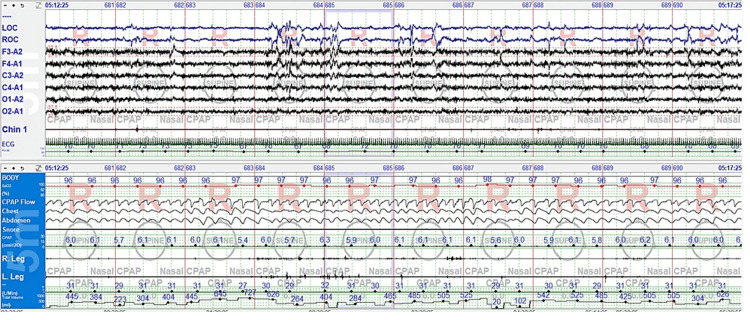
REM sleep with CPAP 6 cmH₂O on titration study: five-minute epoch REM: Rapid eye movement; CPAP: Continuous positive airway pressure.

A subsequent continuous positive airway pressure (CPAP) titration study was performed while the patient remained on the same medication regimen as during the diagnostic study. CPAP was adjusted between 5 and 7 cmH₂O, with 6 cmH₂O effectively eliminating both obstructive and central respiratory events (Figure 3). The residual AHI at 6 cmH₂O was 1.3/hr, snoring was eliminated, and the total arousal index was reduced to 0.6/hr.

**Figure 4:**
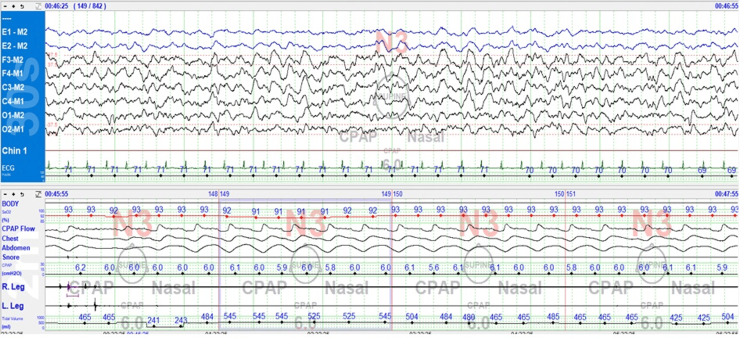
N3 sleep with CPAP 6 cmH₂O: Alpha-delta pattern is abolished, 30-second epoch CPAP: Continuous positive airway pressure.

Remarkably, CPAP intervention also resolved the alpha-delta sleep intrusions (Figure 4), suggesting a significant relationship between the patient's sleep-disordered breathing and sleep architecture disturbances. Sleep architecture showed a slow-wave sleep rebound (N3: 41% vs. 21% on the diagnostic study) at the expense of REM sleep, with delayed REM onset and reduced REM sleep duration (REM: 8.5% vs. 24% in the diagnostic study). No central events were seen during either NREM or REM sleep. Similar to the diagnostic study, the patient remained in the supine position throughout the titration study.

To exclude the underlying neurological causes of central apneas, additional investigations were performed, including a brain MRI, which was normal, and an electroencephalogram in the awake and drowsy states, which was also normal, with no seizures or epileptiform discharges.

The patient had a virtual clinic visit one week after CPAP initiation and reported good adherence (average of six hours per night), with significant improvement in sleep quality and daytime alertness. The patient was subsequently lost to follow-up and could not be reached for further evaluation.

## Discussion

The predominance of central apneas during REM sleep in this patient taking tramadol and pregabalin challenges the conventional understanding of CSA distribution and suggests medication-specific effects on respiratory control.

Given the unusual predominance of central apneas during REM sleep, differential considerations included idiopathic central sleep apnea, medication-induced CSA, and potential underlying neurological causes. However, the patient’s history of chronic opioid and gabapentinoid use, in combination with normal neurological investigations, strongly supported a medication-related etiology for the CSAs.

As previously described, CSA typically occurs during NREM sleep, likely due to differences in chemosensitivity between sleep stages. REM sleep involves reduced chemosensitivity, irregular breathing patterns, and significant hypoventilation, with minute ventilation being lowest during REM sleep. Despite the reduced ventilation and irregular patterns in REM, central apneas are rarely observed in this stage, particularly in OSA patients [2,9]. This makes this patient's REM-predominant pattern particularly noteworthy.

With only a few published case reports, REM-predominant central sleep apnea is uncommon. Reported examples include idiopathic cases, such as in an obese patient with co-existing OSA [10], in association with periodic leg movements [11], and in patients without clear comorbidities, such as REM-related behavior disorder [12,13]. Other cases have implicated neurological etiologies, including bulbar infarction [14] and a possible association with Borrelia burgdorferi infection, although definitive evidence of neuroborreliosis was lacking [15]. This case adds to this limited body of literature and represents the first documented instance of REM-predominant CSA in the context of concurrent tramadol and pregabalin use, suggesting a potential role for medication-induced modulation of REM-related respiratory control.

Opioids are known to induce CSA by reducing respiratory drive and altering CO₂ sensitivity [16]. While potent μ-opioid agonists typically affect the pre-Bötzinger complex and other respiratory control centers, tramadol's unique dual mechanism may contribute to a different pattern of ventilatory control, particularly during REM sleep. Recent evidence suggests that opioid-gabapentinoid combination therapy could be associated with an increased risk of central nervous system (CNS) depression and respiratory depression compared to opioid therapy alone [17], potentially contributing to the unusual pattern of central apneas observed in this patient. The patient reported symptom onset "more than a year ago" while having been on tramadol and pregabalin for approximately two years. Although the precise timeline remains unclear, the symptom development during ongoing medication use supports consideration of medication-induced etiology.

Opioids may exacerbate OSA by diminishing neural signaling to upper airway muscles that maintain pharyngeal patency. However, randomized controlled trials have revealed significant individual variation in response rather than consistent worsening of OSA with opioid use [5]. Meta-analysis data show a significantly increased risk of respiratory depression (OR 1.71) when opioids are combined with gabapentinoids [17], suggesting that the synergistic effects of tramadol and pregabalin may compound central ventilatory depression through complementary mechanisms. In REM sleep, a state already characterized by diminished ventilatory drive, this synergistic interaction likely contributed to the complex presentation of severe OSA and REM-predominant central events by affecting both central respiratory control and upper airway muscle function. However, the precise mechanisms underlying this synergy across different sleep stages remain unclear and warrant further investigation.

In this case, the resolution of the alpha-delta pattern with CPAP therapy suggests a potential mechanistic link between sleep-disordered breathing and alpha-delta sleep. While alpha-delta sleep is commonly associated with chronic pain conditions [7], this patient's alpha-delta pattern resolved completely with CPAP therapy. This improvement likely results from the elimination of respiratory-related arousals, improved oxygenation, and stabilization of sleep continuity. To the best of our knowledge, this is a rarely reported case documenting the resolution of alpha-delta sleep with CPAP therapy alone. The finding suggests that in some patients, sleep-disordered breathing might be a primary contributor to alpha-delta sleep as observed in this patient.

## Conclusions

This case is one of the rare documented instances of medication-associated REM-predominant CSA with concurrent alpha-delta sleep pattern resolution through CPAP therapy, illustrating complex interactions between medications, sleep architecture, and breathing regulation. This rare clinical entity in a patient taking tramadol and pregabalin was successfully managed with CPAP therapy, highlighting the need for comprehensive medication review in atypical sleep-disordered breathing and the potential for sleep architecture improvement with appropriate treatment. Limitations include the inability to establish precise temporal causality due to imprecise patient recall, lack of long-term CPAP compliance data and clinical follow-up due to patient loss to follow-up, and absence of medication-free polysomnographic data. Future research should investigate whether REM-predominant CSA represents a distinct drug-induced endotype and whether alpha-delta patterns serve as EEG markers of sleep disruption in untreated OSA or arousal burden in medicated individuals.
